# 1-(4-Bromo­phen­yl)-3-(2-thienylcarbon­yl)thio­urea

**DOI:** 10.1107/S1600536809038537

**Published:** 2009-09-30

**Authors:** Sohail Saeed, Naghmana Rashid, Rizwan Hussain, Peter G. Jones

**Affiliations:** a Department of Chemistry, Research Complex, Allama Iqbal Open University, Islamabad, Pakistan; b National Engineering and Scientific Commission, PO Box 2801, Islamabad, Pakistan; c Institut für Anorganische und Analytische Chemie, Technische Universität Braunschweig, Postfach 3329, 38023 Braunschweig, Germany

## Abstract

The title compound, C_12_H_9_BrN_2_OS_2_, consists of two planar parts, *viz*. the thio­phene ring including all substituents (r.m.s. deviation 0.007 Å) and the benzene ring including the respective substituents as well as the thione group (r.m.s. deviation 0.05 Å). The inter­planar angle is 18.84 (6)°. An intra­molecular C_phen­yl_—N—H⋯OC hydrogen bond is observed. The three-dimensional packing involves three types of inter­actions, *viz*. N—H⋯S, C—H⋯S (× 2) and Br⋯S [3.6924 (6) Å].

## Related literature

For general background to the chemistry of thio­urea derivatives, see: Choi *et al.* (2008 ▶); Jones *et al.* (2008 ▶); Su *et al.* (2006 ▶). For related structures, see: Saeed *et al.* (2008*a* ▶,*b* ▶,*c* ▶); Yunus *et al.* (2008 ▶). For the cytotoxicity and genotoxicity of anticancer drugs to normal cells in cancer therapy, see: Aydemir & Bilaloglu (2003 ▶).
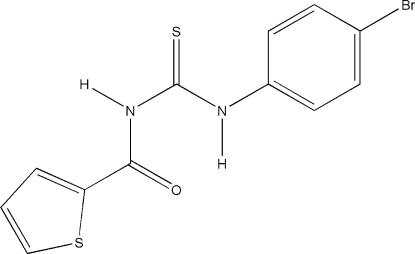


## Experimental

### 

#### Crystal data

C_12_H_9_BrN_2_OS_2_
*M* *_r_* = 341.24
Monoclinic, 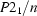

*a* = 13.1483 (6) Å
*b* = 4.4263 (2) Å
*c* = 22.671 (1) Å
β = 90.412 (5)°
*V* = 1319.4 (1) Å^3^
*Z* = 4
Cu *K*α radiation
μ = 7.12 mm^−1^
*T* = 100 K
0.15 × 0.05 × 0.02 mm

#### Data collection

Oxford Diffraction Xcalibur Nova A diffractometer
Absorption correction: multi-scan (*CrysAlis Pro*; Oxford Diffraction, 2009 ▶) *T*_min_ = 0.558, *T*_max_ = 1.000
20285 measured reflections
2712 independent reflections
2438 reflections with *I* > 2σ(*I*)
*R*_int_ = 0.040

#### Refinement

*R*[*F* ^2^ > 2σ(*F* ^2^)] = 0.025
*wR*(*F* ^2^) = 0.067
*S* = 1.06
2712 reflections
171 parameters
H atoms treated by a mixture of independent and constrained refinement
Δρ_max_ = 0.42 e Å^−3^
Δρ_min_ = −0.36 e Å^−3^

### 

Data collection: *CrysAlis Pro* (Oxford Diffraction, 2009 ▶); cell refinement: *CrysAlis Pro*; data reduction: *CrysAlis Pro*; program(s) used to solve structure: *SHELXS97* (Sheldrick, 2008 ▶); program(s) used to refine structure: *SHELXL97* (Sheldrick, 2008 ▶); molecular graphics: *XP* (Siemens, 1994 ▶); software used to prepare material for publication: *SHELXL97*.

## Supplementary Material

Crystal structure: contains datablocks I, global. DOI: 10.1107/S1600536809038537/im2139sup1.cif

Structure factors: contains datablocks I. DOI: 10.1107/S1600536809038537/im2139Isup2.hkl

Additional supplementary materials:  crystallographic information; 3D view; checkCIF report

## Figures and Tables

**Table 1:** Hydrogen-bond geometry (Å, °)

*D*—H⋯*A*	*D*—H	H⋯*A*	*D*⋯*A*	*D*—H⋯*A*
N1—H01⋯S2^i^	0.85 (3)	2.74 (3)	3.5625 (16)	163 (2)
N2—H02⋯O	0.85 (3)	1.89 (3)	2.624 (2)	144 (2)
C9—H9⋯S1^ii^	0.95	2.89	3.704 (2)	144
C2—H2⋯S2^i^	0.95	2.76	3.3193 (18)	119

Symmetry codes: (i) 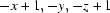; (ii) 
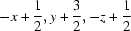
.

**Fig. 1.**
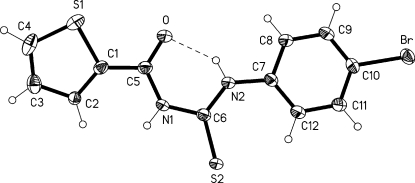
Thermal ellipsoid plot (50% probability level) of the title compound.

**Fig. 2.**
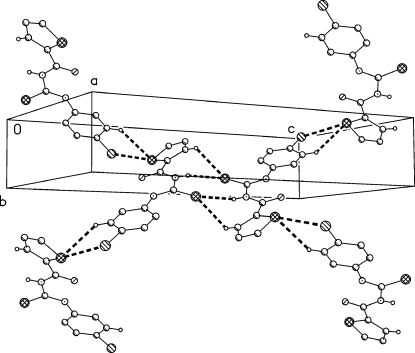
Packing diagram showing the four independent contacts (dashed bonds) N—H···S, C—H···S (× 2), S···Br (see text).

## supplementary crystallographic information

### Comment

The development of new antimicrobial and anticancer therapeutic agents is one of the fundamental goals in medicinal chemistry. Cytotoxicity and genotoxicity of anticancer drugs to normal cells are major problems in cancer therapy and engender the risk of inducing secondary malignancy (Aydemir *et al.*, 2003). A dose of an anticancer drug sufficient to kill tumor cells is often toxic to the normal tissue and leads to many side effects, which, in turn, limit the efficacy of treatment. In recent years, there has been a concerted search for novel selective antitumor agents that lack many of the unpleasant side effects of conventional agents. Thiourea and its derivatives have found extensive applications in the field of medicine, agriculture and analytical chemistry. They are known to exhibit a wide variety of biological activities such as antiviral, anti-bacterial, antifungal, anticancer, antitubercular, herbicidal and insecticidal effects, and also constitute some epoxy resin curing agents containing amino functional groups (Saeed *et al.*, 2008*a*,*b*,*c*). They have found broad areas of application *e.g.* in anion recognition, nonlinear optics and catalysis, and also display good coordination ability (Choi *et al.*, 2008; Jones *et al.*, 2008; Su *et al.*, 2006). As part of our research on thiourea coordination chemistry, we are interested in the study of the influence of non-covalent interactions, especially hydrogen bonds and π-π stacking interactions, on the coordination modes of benzothiazoles bearing the 4-nitrobenzoylthiourea group with transition metal ions. Such coordination compounds of thiourea have been studied for various biological systems in terms of their antibacterial, antifungal and anticancer activities (Yunus *et al.*, 2008).The importance of such work lies in the possibility that the next generation of thiourea derivatives might be more efficacious as antimicrobial and anticancer agents. However, a thorough investigation of their structure, activity and stability under biological conditions is required. These detailed investigations could be helpful in designing more potent antimicrobial and anticancer agents for therapeutic use. The condensation of acyl/aroyl thiocyanates with primary amines affords 1,3-disubstituted thioureas in excellent yields in a single step. In the present paper, the crystal structure of the title compound is reported.

The molecule (Fig. 1) consists of two planar parts: the thiophene ring plus C5 (r.m.s. deviation 0.007 Å) and the phenyl ring plus Br,N2,C6,S2 (0.05 Å), which subtend an interplanar angle of 18.84 (6)°. An intramolecular hydrogen bond N2—H02···O is observed.

The molecular packing is determined by four intermolecular contacts, each of which involves one or other of the sulfur atoms: a surprisingly long classical H bond N1—H01···S2, two weak C—H···S interactions (Table 1) and an interaction C1—S1···Br—C10 with S1···Br 3.6924 (6) Å, C1—S1···Br 165.89 (7) ° and S1···Br—C10 90.51 (6)° (operator for Br and C10: -*x* + 1/2, *y* - 3/2, -*z* + 1/2). The combined effect is to create a three-dimensional pattern, a small part of which is shown in Fig. 2.

### Experimental

A mixture of ammonium thiocyanate (26 mmol) and 2-thiophene carbonyl chloride (26 mmol) in anhydrous acetone (60 ml) was stirred for 45 min. 2-Bromoaniline (26 mmol) was added and the reaction mixture was refluxed for 2 h. After cooling, the reaction mixture was poured into acidified cold water. The resulting dark yellow solid was filtered and washed with cold acetone. The title compound (I) was obtained as colourless needles and laths of several mm length by recrystallization of the solid from ethyl acetate. These tended to split lengthwise when cut, but eventually a fragment suitable for X-ray structure analysis was found.

### Refinement

NH H atoms were refined freely. Other H atoms were placed in calculated positions and refined using a riding model with C—H 0.95 Å; These hydrogen *U* values were fixed at 1.2 × *U*(eq) of the parent atom. Data are 99.4% complete to 2θ 145°.

### Crystal data

**Table:**

C_12_H_9_BrN_2_OS_2_	*F*(000) = 680
*M**_r_* = 341.24	*D*_x_ = 1.718 Mg m^−^^3^
Monoclinic, *P*2_1_/*n*	Melting point: 389 K
Hall symbol: -P 2yn	Cu *K*α radiation, λ = 1.54184 Å
*a* = 13.1483 (6) Å	Cell parameters from 12273 reflections
*b* = 4.4263 (2) Å	θ = 3.4–75.7°
*c* = 22.671 (1) Å	µ = 7.12 mm^−^^1^
β = 90.412 (5)°	*T* = 100 K
*V* = 1319.4 (1) Å^3^	Needle, colourless
*Z* = 4	0.15 × 0.05 × 0.02 mm

### Data collection

**Table:**

Oxford Diffraction Xcalibur Nova A diffractometer	2712 independent reflections
Radiation source: Nova (Cu) X-ray Source	2438 reflections with *I* > 2σ(*I*)
mirror	*R*_int_ = 0.040
Detector resolution: 10.3543 pixels mm^-1^	θ_max_ = 75.9°, θ_min_ = 3.9°
ω scans	*h* = −16→16
Absorption correction: multi-scan (*CrysAlis PRO*; Oxford Diffraction, 2009)	*k* = −5→4
*T*_min_ = 0.558, *T*_max_ = 1.000	*l* = −28→27
20285 measured reflections	

### Refinement

**Table:**

Refinement on *F*^2^	Primary atom site location: structure-invariant direct methods
Least-squares matrix: full	Secondary atom site location: difference Fourier map
*R*[*F*^2^ > 2σ(*F*^2^)] = 0.025	Hydrogen site location: inferred from neighbouring sites
*wR*(*F*^2^) = 0.067	H atoms treated by a mixture of independent and constrained refinement
*S* = 1.06	*w* = 1/[σ^2^(*F*_o_^2^) + (0.0399*P*)^2^ + 0.686*P*] where *P* = (*F*_o_^2^ + 2*F*_c_^2^)/3
2712 reflections	(Δ/σ)_max_ = 0.002
171 parameters	Δρ_max_ = 0.42 e Å^−^^3^
0 restraints	Δρ_min_ = −0.36 e Å^−^^3^

### Special details

**Table:**

Geometry. All e.s.d.'s (except the e.s.d. in the dihedral angle between two l.s. planes) are estimated using the full covariance matrix. The cell e.s.d.'s are taken into account individually in the estimation of e.s.d.'s in distances, angles and torsion angles; correlations between e.s.d.'s in cell parameters are only used when they are defined by crystal symmetry. An approximate (isotropic) treatment of cell e.s.d.'s is used for estimating e.s.d.'s involving l.s. planes.Short contact:3.6924 (0.0006) S1 - Br_$3 165.89 (0.07) C1 - S1 - Br_$3 90.51 (0.06) S1 - Br_$3 - C10_$3 Operator for generating equivalent atoms: $3 - *x* + 1/2, *y* - 3/2, -*z* + 1/2Least-squares planes (*x*,*y*,*z* in crystal coordinates) and deviations from them (* indicates atom used to define plane)- 2.2923 (0.0090) *x* + 3.4843 (0.0018) *y* + 13.4394 (0.0126) *z* = 3.7714 (0.0071)* -0.0064 (0.0017) C1 * -0.0022 (0.0013) C2 * -0.0022 (0.0014) C3 * 0.0106 (0.0014) C4 * -0.0078 (0.0010) S1 * 0.0079 (0.0011) C5Rms deviation of fitted atoms = 0.0069- 5.4364 (0.0073) *x* + 3.6780 (0.0010) *y* + 8.5062 (0.0060) *z* = 1.0213 (0.0056)Angle to previous plane (with approximate e.s.d.) = 18.84 (0.06)* -0.0768 (0.0015) C6 * 0.0657 (0.0011) S2 * 0.0104 (0.0018) C7 * 0.0477 (0.0017) C8 * 0.0508 (0.0017) C9 * 0.0153 (0.0017) C10 * 0.0055 (0.0018) C11 * 0.0033 (0.0020) C12 * -0.0663 (0.0010) Br * -0.0558 (0.0015) N2 - 0.2202 (0.0028) C5 - 0.1410 (0.0028) O -0.2122 (0.0021) N1Rms deviation of fitted atoms = 0.0479
Refinement. Refinement of *F*^2^ against ALL reflections. The weighted *R*-factor *wR* and goodness of fit *S* are based on *F*^2^, conventional *R*-factors *R* are based on *F*, with *F* set to zero for negative *F*^2^. The threshold expression of *F*^2^ > σ(*F*^2^) is used only for calculating *R*-factors(gt) *etc*. and is not relevant to the choice of reflections for refinement. *R*-factors based on *F*^2^ are statistically about twice as large as those based on *F*, and *R*- factors based on ALL data will be even larger.

### Fractional atomic coordinates and isotropic or equivalent isotropic displacement parameters (Å^2^)

**Table:**

	*x*	*y*	*z*	*U*_iso_*/*U*_eq_	
Br	0.629471 (15)	0.87392 (5)	0.136702 (8)	0.02773 (9)	
S1	0.12627 (4)	−0.47575 (13)	0.42493 (2)	0.03066 (13)	
S2	0.57481 (4)	0.15778 (13)	0.42693 (2)	0.02881 (13)	
O	0.27346 (11)	−0.1628 (4)	0.34866 (6)	0.0306 (3)	
N1	0.40397 (12)	−0.1458 (4)	0.41634 (7)	0.0213 (3)	
H01	0.4194 (19)	−0.180 (6)	0.4521 (12)	0.031 (7)*	
N2	0.44284 (13)	0.1420 (4)	0.33512 (7)	0.0238 (4)	
H02	0.385 (2)	0.075 (6)	0.3245 (11)	0.032 (7)*	
C1	0.25426 (14)	−0.4373 (5)	0.43690 (8)	0.0228 (4)	
C2	0.28750 (14)	−0.6036 (4)	0.48600 (8)	0.0191 (4)	
H2	0.3558	−0.6113	0.4998	0.023*	
C3	0.20512 (17)	−0.7586 (5)	0.51212 (9)	0.0303 (4)	
H3	0.2122	−0.8857	0.5457	0.036*	
C4	0.11538 (17)	−0.7072 (5)	0.48445 (11)	0.0340 (5)	
H4	0.0527	−0.7916	0.4969	0.041*	
C5	0.31026 (14)	−0.2406 (5)	0.39652 (8)	0.0226 (4)	
C6	0.47172 (14)	0.0541 (5)	0.38914 (8)	0.0215 (4)	
C7	0.49109 (15)	0.3288 (4)	0.29299 (8)	0.0213 (4)	
C8	0.43466 (15)	0.3754 (5)	0.24114 (9)	0.0251 (4)	
H8	0.3683	0.2922	0.2375	0.030*	
C9	0.47486 (15)	0.5415 (5)	0.19539 (9)	0.0268 (4)	
H9	0.4365	0.5732	0.1603	0.032*	
C10	0.57114 (15)	0.6608 (4)	0.20115 (8)	0.0228 (4)	
C11	0.62707 (15)	0.6225 (5)	0.25232 (9)	0.0279 (4)	
H11	0.6928	0.7100	0.2559	0.033*	
C12	0.58713 (15)	0.4557 (5)	0.29867 (9)	0.0293 (4)	
H12	0.6254	0.4288	0.3340	0.035*	

### Atomic displacement parameters (Å^2^)

**Table:**

	*U*^11^	*U*^22^	*U*^33^	*U*^12^	*U*^13^	*U*^23^
Br	0.02733 (13)	0.03447 (14)	0.02145 (12)	0.00217 (8)	0.00351 (8)	0.00748 (8)
S1	0.0204 (2)	0.0323 (3)	0.0393 (3)	−0.0031 (2)	−0.00143 (19)	−0.0057 (2)
S2	0.0253 (2)	0.0430 (3)	0.0181 (2)	−0.0118 (2)	−0.00757 (18)	0.00732 (19)
O	0.0251 (7)	0.0443 (9)	0.0222 (7)	−0.0074 (6)	−0.0074 (6)	0.0054 (6)
N1	0.0211 (8)	0.0283 (9)	0.0145 (8)	−0.0019 (6)	−0.0039 (6)	0.0029 (6)
N2	0.0208 (8)	0.0319 (9)	0.0187 (8)	−0.0042 (7)	−0.0045 (6)	0.0038 (7)
C1	0.0193 (8)	0.0278 (10)	0.0212 (9)	−0.0023 (8)	0.0005 (7)	−0.0055 (8)
C2	0.0217 (9)	0.0198 (9)	0.0159 (8)	−0.0038 (7)	0.0010 (7)	−0.0037 (7)
C3	0.0391 (11)	0.0255 (10)	0.0266 (10)	−0.0025 (9)	0.0095 (8)	−0.0036 (9)
C4	0.0286 (10)	0.0260 (11)	0.0477 (13)	−0.0049 (9)	0.0173 (9)	−0.0085 (10)
C5	0.0207 (8)	0.0272 (10)	0.0199 (9)	−0.0008 (8)	−0.0020 (7)	−0.0027 (8)
C6	0.0213 (8)	0.0258 (10)	0.0174 (8)	0.0004 (7)	−0.0024 (7)	0.0002 (7)
C7	0.0230 (9)	0.0247 (10)	0.0162 (8)	0.0002 (7)	−0.0009 (7)	0.0014 (7)
C8	0.0223 (9)	0.0320 (11)	0.0210 (9)	−0.0031 (8)	−0.0047 (7)	0.0021 (8)
C9	0.0290 (10)	0.0331 (11)	0.0184 (9)	0.0002 (9)	−0.0053 (7)	0.0041 (8)
C10	0.0264 (9)	0.0232 (10)	0.0190 (9)	0.0034 (7)	0.0027 (7)	0.0035 (7)
C11	0.0222 (9)	0.0376 (12)	0.0238 (10)	−0.0027 (8)	−0.0029 (8)	0.0053 (8)
C12	0.0253 (10)	0.0411 (12)	0.0212 (9)	−0.0027 (9)	−0.0052 (7)	0.0077 (9)

### Geometric parameters (Å, °)

**Table:**

Br—C10	1.9050 (19)	C7—C12	1.387 (3)
S1—C4	1.701 (3)	C7—C8	1.401 (3)
S1—C1	1.7111 (19)	C8—C9	1.379 (3)
S2—C6	1.6629 (19)	C9—C10	1.377 (3)
O—C5	1.234 (2)	C10—C11	1.380 (3)
N1—C5	1.374 (2)	C11—C12	1.390 (3)
N1—C6	1.402 (3)	N1—H01	0.85 (3)
N2—C6	1.338 (2)	N2—H02	0.85 (3)
N2—C7	1.417 (3)	C2—H2	0.9500
C1—C2	1.402 (3)	C3—H3	0.9500
C1—C5	1.465 (3)	C4—H4	0.9500
C2—C3	1.415 (3)	C8—H8	0.9500
C3—C4	1.352 (3)	C9—H9	0.9500
			
C4—S1—C1	91.28 (10)	C11—C10—Br	119.39 (15)
C5—N1—C6	128.33 (16)	C10—C11—C12	119.92 (19)
C6—N2—C7	131.42 (17)	C7—C12—C11	119.56 (18)
C2—C1—C5	130.75 (17)	C5—N1—H01	117.8 (18)
C2—C1—S1	111.98 (14)	C6—N1—H01	112.6 (18)
C5—C1—S1	117.26 (14)	C6—N2—H02	113.9 (18)
C1—C2—C3	110.59 (17)	C7—N2—H02	114.7 (18)
C4—C3—C2	113.1 (2)	C1—C2—H2	124.7
C3—C4—S1	113.04 (16)	C3—C2—H2	124.7
O—C5—N1	123.18 (18)	C4—C3—H3	123.5
O—C5—C1	121.29 (17)	C2—C3—H3	123.5
N1—C5—C1	115.51 (16)	C3—C4—H4	123.5
N2—C6—N1	114.16 (16)	S1—C4—H4	123.5
N2—C6—S2	128.06 (16)	C9—C8—H8	119.8
N1—C6—S2	117.77 (13)	C7—C8—H8	119.8
C12—C7—C8	119.64 (18)	C10—C9—H9	120.3
C12—C7—N2	125.84 (17)	C8—C9—H9	120.3
C8—C7—N2	114.50 (17)	C10—C11—H11	120.0
C9—C8—C7	120.39 (18)	C12—C11—H11	120.0
C10—C9—C8	119.34 (18)	C7—C12—H12	120.2
C9—C10—C11	121.11 (18)	C11—C12—H12	120.2
C9—C10—Br	119.48 (15)		
			
C4—S1—C1—C2	0.46 (16)	C5—N1—C6—N2	−5.5 (3)
C4—S1—C1—C5	−178.78 (16)	C5—N1—C6—S2	173.38 (16)
C5—C1—C2—C3	179.2 (2)	C6—N2—C7—C12	3.5 (4)
S1—C1—C2—C3	0.1 (2)	C6—N2—C7—C8	−178.4 (2)
C1—C2—C3—C4	−0.8 (3)	C12—C7—C8—C9	1.3 (3)
C2—C3—C4—S1	1.1 (2)	N2—C7—C8—C9	−177.00 (19)
C1—S1—C4—C3	−0.92 (18)	C7—C8—C9—C10	0.1 (3)
C6—N1—C5—O	2.2 (3)	C8—C9—C10—C11	−1.4 (3)
C6—N1—C5—C1	−176.30 (18)	C8—C9—C10—Br	177.04 (16)
C2—C1—C5—O	164.5 (2)	C9—C10—C11—C12	1.4 (3)
S1—C1—C5—O	−16.4 (3)	Br—C10—C11—C12	−177.09 (17)
C2—C1—C5—N1	−17.0 (3)	C8—C7—C12—C11	−1.3 (3)
S1—C1—C5—N1	162.10 (14)	N2—C7—C12—C11	176.8 (2)
C7—N2—C6—N1	−177.11 (19)	C10—C11—C12—C7	0.0 (3)
C7—N2—C6—S2	4.2 (3)		

### Hydrogen-bond geometry (Å, °)

**Table:**

*D*—H···*A*	*D*—H	H···*A*	*D*···*A*	*D*—H···*A*
N1—H01···S2^i^	0.85 (3)	2.74 (3)	3.5625 (16)	163 (2)
N2—H02···O	0.85 (3)	1.89 (3)	2.624 (2)	144 (2)
C9—H9···S1^ii^	0.95	2.89	3.704 (2)	144
C2—H2···S2^i^	0.95	2.76	3.3193 (18)	119

Symmetry codes: (i) −*x*+1, −*y*, −*z*+1; (ii) −*x*+1/2, *y*+3/2, −*z*+1/2.

## References

[bb1] Aydemir, N. & Bilaloglu, R. (2003). *Mutat. Res.* **537**, 43–51. 10.1016/s1383-5718(03)00049-4 12742506

[bb2] Choi, M. K., Kim, H. N., Choi, H. J., Yoon, J. & Hyun, M. H. (2008). *Tetrahedron Lett.* **49**, 4522–4525.

[bb3] Jones, C. E., Turega, S. M., Clarke, M. L. & Philp, D. (2008). *Tetrahedron Lett.* **49**, 4666–4669.

[bb4] Oxford Diffraction (2009). *CrysAlis Pro* Oxford Diffraction Ltd, Yarnton, England.

[bb5] Saeed, S., Bhatti, M. H., Tahir, M. K. & Jones, P. G. (2008*a*). *Acta Cryst.* E**64**, o1369. 10.1107/S1600536808017868 PMC2961839 21202987

[bb6] Saeed, S., Bhatti, M. H., Yunus, U. & Jones, P. G. (2008*b*). *Acta Cryst.* E**64**, o1485. 10.1107/S1600536808017856 PMC2962115 21203197

[bb7] Saeed, S., Bhatti, M. H., Yunus, U. & Jones, P. G. (2008*c*). *Acta Cryst.* E**64**, o1566. 10.1107/S1600536808022095 PMC2962175 21203269

[bb8] Sheldrick, G. M. (2008). *Acta Cryst.* A**64**, 112–122. 10.1107/S0108767307043930 18156677

[bb9] Siemens (1994). *XP* Siemens Analytical X-ray Instruments Inc., Madison, Wisconsin, USA.

[bb10] Su, B.-Q., Liu, G.-L., Sheng, L., Wang, X.-Q. & Xian, L. (2006). *Phosphorus Sulfur Silicon*, **181**, 745–750.

[bb11] Yunus, U., Tahir, M. K., Bhatti, M. H., Ali, S. & Wong, W.-Y. (2008). *Acta Cryst.* E**64**, o20. 10.1107/S160053680706134X PMC2914982 21200765

